# Measuring interoperable EHR adoption and maturity: a Canadian example

**DOI:** 10.1186/s12911-016-0247-x

**Published:** 2016-01-25

**Authors:** Bobby Gheorghiu, Simon Hagens

**Affiliations:** Canada Health Infoway - Inforoute Santé du Canada, 150 King St. W., Ste 1300, Toronto, ON M5H 1J9, Canada

**Keywords:** Health information exchange, Electronic health record, Technology adoption, Maturity of use, Change management, Health information technology

## Abstract

**Background:**

An interoperable electronic health record is a secure consolidated record of an individual’s health history and care, designed to facilitate authorized information sharing across the care continuum.  Each Canadian province and territory has implemented such a system and for all, measuring adoption is essential to understanding progress and optimizing use in order to realize intended benefits.

**Results:**

About 250,000 health professionals—approximately half of Canada’s anticipated potential physician, nurse, pharmacist, and administrative users—indicated that they electronically access data, such as those found in provincial/territorial lab or drug information systems, in 2015.  Trends suggest further growth as maturity of use increases.

**Conclusions:**

There is strong interest in health information exchange through the iEHR in Canada, and continued growth in adoption is expected. Central to managing the evolution of digital health is access to robust data about who is using solutions, how they are used, where and when.  Stakeholders such as government, program leads, and health system administrators must critically assess progress and achievement of benefits, to inform future strategic and operational decisions.

## Background

Efforts to build an interoperable electronic health record (iEHR) have been underway across Canada for many years. While deployment progress and rates of user adoption vary across provinces and territories, the initiative has passed its tipping point and uptake is now increasing rapidly. The potential benefits of the iEHR are substantial—improved quality of care, system efficiencies, improved access to care and use of health data to better manage the health system and facilitate ground-breaking research. An iEHR is a secure, integrated view of a person’s medical records from all systems in the network; it provides a comprehensive view of a patient’s medical history [1]. Typically, it integrates data from diagnostic imaging systems, laboratory information systems (LIS) as well as drug information systems (DIS), to provide a longitudinal view of a patient’s clinical history. As such, it is a similar concept to that of a Health Information Exchange (HIE) [2]. Across Canada, iEHRs are at various stages of implementation and maturity and have evolved according to provincial/territorial strategies and priorities.

The iEHR acts as a complement to point of service systems like electronic medical records (EMR) in physician offices or clinical information systems (CIS) in hospitals. Regular measurement of adoption and maturity for these technologies has made progress easy to follow and manage. For example, in the 2014 National Physician Survey, 77 % of all family physicians reported they do use electronic records to enter and retrieve clinical notes [3]. Canadian hospital scores from the HIMSS Electronic Medical Record Adoption Model (EMRAM) show that while 81 % of hospitals have local Laboratory, Radiology and Pharmacy Systems, only 37 % are more advanced in their use, such as nursing/clinical documentation and clinical decision support [4].

Canada has been an active supporter of activities to monitor and benchmark progress in digital health broadly, such as the work of OECD [5] and the Commonwealth Fund [6]. This work has been important to increase quality and consistency of measurement across countries. The work also surfaces some of the challenges in effectively measuring adoption and has been a driver in Canada to pursue more advanced methods which go beyond common survey based approaches. As the use of social media has proliferated, the monthly active use metric has become an accepted indicator of overall reach and impact for such ubiquitous companies as Facebook and Twitter [7]. Using the same concept to measure digital health adoption can yield similar insights and value.

Emerging statistics around adoption and impacts of iEHRs in Canada confirm the time and continued effort required after initial implementation go-live to enable the realization of intended benefits. With increased attention from various levels of government to create a sustainable healthcare system that meets the needs of its populations, the emphasis on reporting progress related to the availability, adoption, and active use of useful, integrated iEHRs has never been more pressing.

## iEHR deployment in Canada

**Fig. 1:**
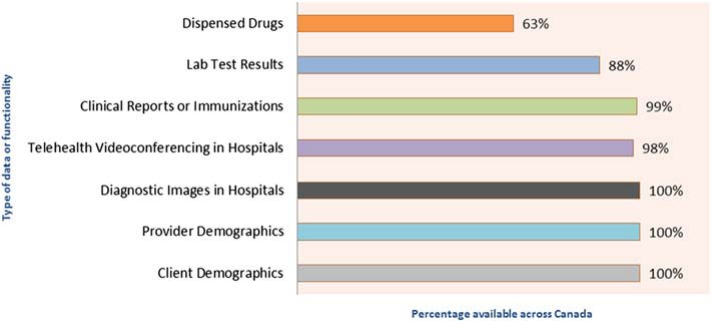
Digitization of information for authorized users. Digitization does not measure the extent of use by providers, but rather the information and systems that are in place

Canada Health Infoway, an independent, not-for-profit organization funded by the federal government, tracks several different metrics to measure progress toward a comprehensive iEHR within each province and territory. One such metric focuses on the availability of iEHR data. “Available” means that the data have been electronically stored in a database accessible by authorized health care professionals. Availability is tracked for each of six core iEHR domains: Client Registry, Provider Registry, Diagnostic Imaging (DI), Laboratory Test Results, Drugs Dispensed, and Clinical Reports/Immunizations. Provincial and territorial rates from each domain (weighted by population) are aggregated to arrive at a pan-Canadian value. The graphic in Fig. 1 shows the domain-specific availability rates as of March 31, 2015 [8].

Access to the iEHR for authorized clinical and administrative users is made available either through integration of various point-of-care systems such as CIS and EMR or through web-enabled viewers that extract relevant patient data from various clinical databases and present the information in a coherent, easy-to-digest manner. Table 1 provides descriptions of the jurisdictions that currently have an iEHR available for use. Availability is a valuable indicator for tracking the completion of iEHR implementation projects and for determining which functions are available to clinicians and other health system users. However, it is not a reliable indicator of technology adoption since it does not specify the frequency of use nor the types and number of users.

**Table 1:** iEHR deployment statistics as of January 31, 2015

Jurisdiction	Go-live	Clinical domains currently live	Clinical settings
Newfoundland	2014	Lab, drug profile	Hospitals
Prince Edward Island	2008	Lab, drug, DI	Hospitals, primary care
Nova Scotia	2010	Lab, DI, clinical reports	Hospitals
New Brunswick	2010	Lab, DI, cardiology reports	Hospitals
Quebec	2013	Lab, drug, DI	Hospitals, primary care
Ontario	2011	Lab, DI, clinical reports, drug profiles	Hospitals (selected regions), primary care
Manitoba	2011	Lab, drug, DI, immunization, clinical reports	Hospitals, primary care
Saskatchewan	2013	Lab, drug	Hospitals, primary care
Alberta	2006	Lab, drug profile, DI, immunization, clinical reports, allergies	Hospitals, pharmacies, primary care, ambulatory
British Columbia	2010	Lab, DI	Hospitals
Northwest Territories	2010	Lab, DI, clinical reports	Hospital, primary care, public health offices
Nunavut	2011	Lab, DI, drug profile, clinical reports	Hospitals

## Methods

### Metrics and data sources

Adoption monitoring for the purpose of demonstrating usage of the iEHR over time is supported by data from surveys of clinicians and patients, usage data from digital health solutions, and operational data sets collected by our partners. Specific definitions of adoption are designed to suit distinct kinds of solutions, and trended over time.

**Fig. 2:**
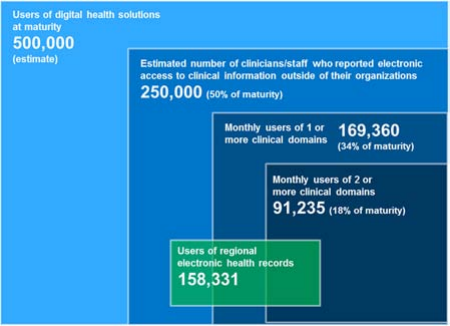
Canadian iEHR user landscape. Over 50 % of doctors, nurses and pharmacists surveyed indicated that they have access to clinical information outside their organization. This includes users of provincial/territorial EHRs (2+ clinical domains), users of single clinical domains (e.g., drug information systems), and users of regional EHRs. In total, this represents about half of all potential EHR users across Canada (estimated at 500,000). ‘Users of digital health solutions at maturity’ assumes 80 % of all physicians, nurses and pharmacists along with an additional 50 % for other clinicians and administrators

Four metrics of adoption (accompanied by a universe estimate) are included in Fig. 2:

- monthly users of 2 or more clinical domains
- monthly users of 1 or more clinical domains
- Users of regional health records
- Clinicians/staff who reported electronic access to clinical information outside of their organization

The data informing the “1 or more” and “2 or more” domain totals (Fig. 2) is collected through a collaborative process with stakeholders such as administrators of provincial/territorial iEHRs, with the goal of obtaining a standardized data set from respective databases. A similar process was carried out in 2015 to obtain regional iEHR user data for the first time. The preferred data set includes:

- Number of active users
- Total logins
- Total transactions
- Transactions per user
- Transactions by user type (physician, nurse, pharmacist administrative, etc.)

The definition of an active user of an iEHR varies by jurisdiction but meets the criteria of at least one system access per month. This definition exists in order to filter out system users who may be registered and have the credentials to use an iEHR but do not use it on a regular basis. Users are health system professionals such as clinicians and administrators who have access to the iEHR via standalone electronic viewers or through integrated point-of-care systems such as CIS or EMR.

Through quality assurance activities, data for each jurisdiction is checked for quality and interpretation issues by comparing reported users against potential user bases, previous data sets, clinician survey data and other sources. Qualitative investigations such as Q & A sessions with the reporting organizations are used to understand definitions and contextual factors. Issues identified are resolved in collaboration with the jurisdictional partner. These metrics are the most robust measures, and the focus for annual trending.

The estimate for “clinicians/staff who reported electronic access” is modelled from nationally representative surveys of physicians (*n* = 10,191, collected April-July 2014) [9], nurses (*n* = 1,690, collected February-March 2014) [10] and pharmacists (*n* = 447, collected February-March 2014) [11]. The respondents to each survey were asked to identify “which of the following electronic tools you use in the care of your patients”, and provided with a bank of options relevant to their practice. Respondents who identified themselves as users of “access to provincial/territorial patient information systems”, lists of “all medications taken by a patient”, or “electronic receipt of laboratory test results from external laboratory/diagnostic imaging”, were classified as “clinicians/staff who reported electronic access” for this analysis. Estimates are generated by applying these response rates to national workforce data for active physicians, nurses, and pharmacists across Canada. To account for users of solutions not from one of the three clinician groups surveyed, the analysis included development of estimates relating to this group. A review of system-generated reports for eight provinces and territories showed an average of 20 % of iEHR viewer users to be administrative and 10 % Allied Health. Clinician interviews generated an estimate of 50:50 ratio of clinical to administrative users in community based physician offices. Based upon these findings, an overall estimate of 30 % additional staff was included in the calculation.

The estimates for use of regional iEHRs are collected primarily through key informant interviews, and are the most preliminary in nature.

### Limitations

Limitations of this iEHR adoption definition and measurement approach include variability in data quality, reporting capability and local context. Data from iEHR solutions may reflect different definitions of user types, transactions, logins, etc. Quality assurance activities are not always able to resolve all discrepancies. Not all jurisdictions are able to contribute the full preferred data set, limiting some analysis, benchmarking and quality assurance. Usage data from the iEHRs does not always capture the full extent of use of the data. For example, in many reported scenarios, iEHR data is imported into a local system in some format or printed and put in a paper chart. In these scenarios, the data is being used by many more clinicians than might be reported in the iEHR solution. This may account for survey reported iEHR adoption which is greater than usage data directly extracted from the iEHRs. When interpreting survey results, it is also very difficult to ascertain exactly which systems are being accessed by clinicians when they indicate that they are able to access electronic patient information from outside their own organization. From a clinician point of view, they have access to the information that they need with the distinction between a regional and provincial/territorial system being less important to their decision-making ability.

## Result

Across Canada, iEHRs are at various stages of implementation and maturity and have evolved according to provincial/territorial strategies and priorities. As of January 2015, all provinces and territories were reporting active users of 2+ clinical domains (note: this is the only measure for which historical usage data is available) [8].

A stacked Venn diagram (Fig. 2) was used to illustrate the heterogeneous nature and usage of these solutions while at the same time presenting a comprehensive view of the iEHR landscape across Canada.

The smallest area in the centre of the diagram represents the number of active users of jurisdiction-wide systems who have access to at least two clinical data domains (e.g., lab, drug, DI, etc.) within a viewer application or point-of-care system.

The next level, monthly users of 1+ domains, adds those users who only have access to one specific clinical domain, for example, public health nurses who have access to a provincial public health surveillance system or retail pharmacists who are accessing a drug information system. Users of regional iEHR systems have access to similar types of information as those who access provincial/territorial-wide systems but are limited to data specific to a geographical or administrative region. Regional system users may also be users of single or multiple domain provincial/territorial systems although the exact overlap is unknown (represented by a partial overlap in Fig. 2).

The estimate of 250,000 users of the iEHR relies on publicly available baseline and survey data and represents clinicians’ perceptions of having the information they need in electronic format. This represents 58 of physicians, 53 of nurses, and 53 % of pharmacists who report access to or are using drug, lab or other iEHR data [9–11]. Administrative and other clinical (e.g., Allied Health) users were estimated to be an additional 30 and 10 % respectively based on data modeled from available information about user type distribution. The potential user base for digital health solutions at maturity, estimated at 500,000 users across Canada, was derived by assuming use by 80 % of all physicians, registered nurses and pharmacists plus an additional 50 % of this total to account for other clinical and support staff. This was deemed to be a conservative estimate that would allow for strategic planning over the next 5–10 years. It is based on multiple theories of technology adoption and diffusion that identify late adopters or “laggards” as the last 15–20 % of the population to adopt a technology [12, 13].

## Discussion: adoption and maturity

Tracking and reporting of adoption data is an important means of determining the spread of digital health solutions and a useful tool towards the realization of intended outcomes and benefits. A literature review of international practices in tracking adoption yielded a number of metrics, which focused on the ability of health care organizations to access or share clinical data. A well-cited example is the HIMSS EMR Adoption Model that scores organizations on the implementation of various clinical applications and capabilities [4]. However, no evidence was found supporting the tracking and reporting of active use of the iEHR (or HIE) on a national scale.

**Fig. 3:**
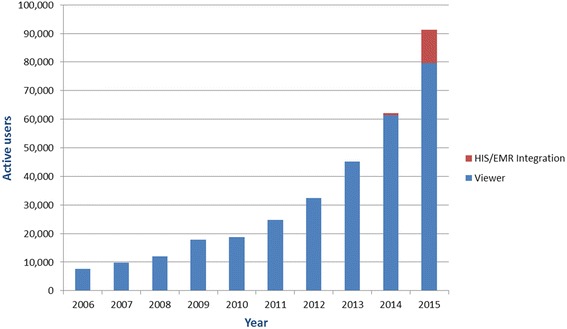
Trending iEHR systems use (2+ clinical domains) by type of access. Figures represent active users with access to two or more integrated provincial data assets (e.g., lab information system, drug information system, diagnostic imaging repository, etc.). Active users have accessed the system a minimum of one time per month. Users of EMR and HIS integrated with 2+ clinical domains are deemed to be active users of the iEHR

Adoption has the potential to accelerate dramatically when iEHR data is integrated into existing point of service systems (such as hospital information systems or EMRs in primary care), or launched in context from a local system. This model is partially responsible for the accelerated growth in active use of provincial/territorial iEHRs (2+ domains) seen in the past two years (Fig. 3). Currently, 13 % of iEHR users are accessing data via integrated point-of-care systems—up from 1 % in the previous year.

**Fig. 4:**
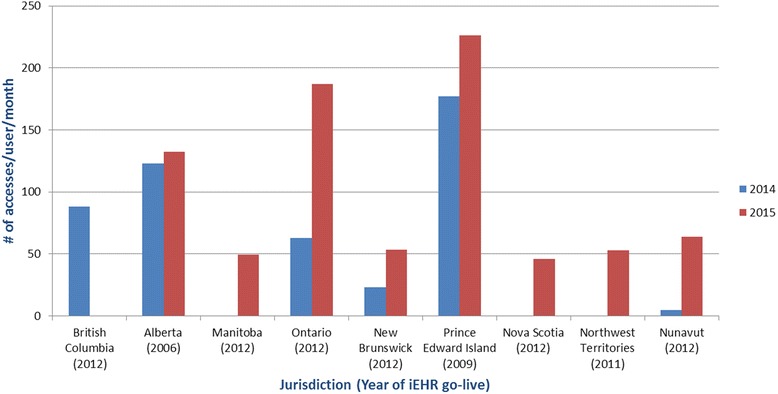
Number of iEHR accesses per user by jurisdiction. Data presented for those jurisdictions and years where access and active user data is available

A different method of gauging system maturity is by how frequently end users are accessing their iEHR. An analysis that maps system accesses against numbers of active users across various jurisdictions across Canada shows a large variation in the number of iEHR accesses per user per month (Fig. 4). The two jurisdictions with the highest frequency of access are also the ones that have been operational the longest, supporting the idea that end users are likely to see more benefits in the use of systems that are more mature.

In Canada, as iEHRs are moving from the deployment stage towards broad clinical adoption, the focus will need to shift towards optimization of these systems to meet clinical and consumer needs. Comparable systems and initiatives internationally, such as HIE in the United States, have demonstrated the electronic health record as a foundation technology for improved utilization of services, improved chronic disease management and more patient-centered care and a powerful source of information to manage the health system [14]. Seventy-six percent of hospitals reported exchanging data with outside health professionals in 2014; up from 62 in 2013 and 41 % in 2008, the year the survey began including this measure [15]. Continuing to build an understanding of this maturity curve and the success factors is critical to benefits realization in the long-term. Expectations must be managed, benefits strategically targeted and success factors identified and addressed. A comprehensive and sustained change management approach (including evaluation and continuous improvement) not only brings users on board, but moves users and organizations up the curve to realize the full potential. As an example, Alberta’s Netcare system started as a small, local pilot project, Alberta Pharmaceutical Information Network (PIN) in 2002, and has continued to expand as a result of the pilot’s demonstrated value and user satisfaction [16]. Twelve years later, it is still growing at a rapid pace as it becomes an indispensable tool for clinicians across the province, and as more and more health service providers, beyond the hospital sector, are accessing and contributing health information.

## Conclusion

The Canadian health care landscape is rapidly changing with examples of innovation and process improvements happening both at the point of care and in large, jurisdiction-wide initiatives. Health care system-wide adoption of iEHRs will likely require support for both approaches. Central to managing the evolution of digital health is access to robust data about who is using solutions, how they are used, where and when. Stakeholders such as government, program leads, and health system administrators must critically assess progress and achievement of benefits, to inform future strategic and operational decisions.

By tracking, analyzing and reporting data regarding the adoption of iEHRs and other digital health technologies, one hopes to bring the required focus and attention necessary for sustaining the momentum of implementation and adoption of iEHR solutions leading to the realization of benefits. In Canada, this tracking has allowed health system leaders to demonstrate the rapid adoption of iEHR investments in recent years, and better understand the drivers required to maintain this trend. Key stakeholders across the system are now better informed and able to share leading practices and lessons learned, and assist in solving areas of challenge.

## Abbreviations

iEHR: Interoperable electronic health record(s)
LIS: Laboratory information system(s)
DIS: Drug information system(s)
HIE: Health information exchange
EMRAM: Electronic Medical Record Adoption Model
DI: Diagnostic imaging
CIS: Clinical information system(s)
EMR: Electronic medical record(s)
